# Trauma and Poor Mental Health in Relation to Economic Status: The Case of Cambodia 35 Years Later

**DOI:** 10.1371/journal.pone.0136410

**Published:** 2015-08-24

**Authors:** Johan Jarl, Elizabeth Cantor-Graae, Thida Chak, Ka Sunbaunat, Charlotte A Larsson

**Affiliations:** 1 Health Economics Unit, Department of Clinical Science, Lund University, Lund, Sweden; 2 Health Economics & Management, Institute of Economic Research, Lund University, Lund, Sweden; 3 Division of Social Medicine and Global Health, Department of Clinical Science, Lund University, Malmö, Sweden; 4 Department of Psychiatry, Faculty of Medicine, University of Health Sciences, Phnom Penh, Cambodia; Univ of Toledo, UNITED STATES

## Abstract

**Background:**

Cambodia is one of the poorest countries in south-east Asia and is still emerging from the events of the Khmer Rouge reign. It has been suggested that the atrocities experienced by the Cambodian population can explain why Cambodia continues to lag behind its neighbours in economic outcomes. The purpose of this study is to investigate whether there is an association between exposure to past trauma and/or current poor mental health and current economic status in Cambodia.

**Method:**

A newly conducted survey performed in two regions (north-west and south-east Cambodia) collected information on trauma exposure, psychiatric symptoms, self-rated health outcomes and socio-economic information for 3200 persons aged 18–60. Economic outcomes were measured as household debt and poverty status and whether the respondent was economically inactive. All models were analysed using logistic regression.

**Results:**

No association was found between high exposure to conflict-related or civilian trauma and any economic outcomes save for a negative association between civilian trauma and poverty in the south-east. Current post-traumatic stress was related solely to poverty status. All other measures of current mental health status, however, were found to be strongly negatively associated with all measures of economic status. Thus, mental health interventions could potentially be utilised in poverty reduction strategies, but greater efficacy is likely to be achieved by targeting current mental health status rather than previous trauma exposure.

## Introduction

Cambodia is not only one of the poorest countries in the world, but also has one of the most troublesome recent histories. However, the country has experienced a very fast growth rate (generally above 5%) since the establishment of political stability in 1991, and especially during the decade following the brief civil war in 1997 [1]. Due to the low starting position, it is difficult to put the achievement into context, but the country performs favourably compared to other post-conflict settings [1]. Cambodia has not only increased gross national income per capita but also has increased life expectancy at birth, living standards, and has experienced a rapid fall in the incidence of poverty. However, around 75% still live below or just above the poverty line [1, 2].

Despite the recent improvements, Cambodia still lags behind its close neighbours in both GNI and growth rate [2], and a large part of the economic improvement could be seen as a peace effect [3]. According to the UN, Cambodia remains one of the least developed economies. It has often been suggested that the atrocities committed during the Khmer Rouge reign can serve as an explanation for Cambodia’s particular situation. In addition to widespread destruction of infrastructure, capital, and still active landmines, the human damage has been substantial both in terms of deceased (especially under five mortality, adult males, educated and rural individuals), and survivors being disabled and/or emotionally affected by the traumatic events [4, 5].

High prevalence rates of war/conflict related trauma have been reported in the Cambodian population [6, 7]. Poor mental health as a consequence of exposure to trauma and structural violence is a well-established finding (e.g. [5, 8]). It would therefore be expected that high rates of post-traumatic stress disorder and other psychiatric disorders would currently be found in Cambodia. Such was the case in Sonis et al.‘s national survey [7] although Cantor-Graae et al. [6] recently found high rates of trauma but only low rates of psychiatric disorders in selected rural areas. Expectations are, nevertheless, that the Cambodian population is more emotionally distressed and has poorer mental health than the populations of its neighbouring countries, such as Laos and Thailand. Given a well-known link between poor mental health and poverty [9], it might appear surprising that Cambodia is not in an even worse economic situation. Still, it is unknown to what extent trauma exposure constitutes an impediment for improved economic well-being, both for the country and the individual. A study on Cambodian refugees in the USA during the 1980s found that individuals who experienced self-reported persistent effects of trauma exposure had an increased likelihood of unemployment as well as higher income, while the actual number of traumas was related to reduced income [10]. Other studies have indicated that Cambodian youths relocated to the USA function well in terms of school grades [11] and generally in education and occupation [12], irrespective of post-traumatic stress disorder. However, a limited sample and a substantially different context make these results difficult to apply to a Cambodian setting. The question thus remains if and to what extent prevalent exposure to potentially traumatic events in Cambodia has negative economic consequences.

A working hypothesis in the current study is that exposure to trauma might have a more global effect on daily life than merely contributing to poor mental health. The argument is that there is a direct effect of trauma on economic status, especially considering Cambodia’s recent history, in addition to the indirect effect through poor mental health. Due to the very high prevalence of trauma in Cambodia [6] especially in adults with direct exposure to the Khmer Rouge era, it is not viable to compare individuals with and without trauma exposure per se. A preferable approach is to examine the potential impact of trauma on current economic status in terms of “high” versus “low” exposure to trauma, based on the number of trauma reported. Moreover, due to the number of years that have elapsed since the Khmer Rouge reign, it is reasonable to assume that a more comprehensive assessment of lifetime exposure to trauma would be needed that also includes trauma exposure in civilian life.

The purpose of this study is therefore two-fold: a) to examine the impact of high trauma exposure (conflict- and civilian-related) and/or poor mental health on current economic status (household and individual level), and b) to examine whether recovery time affects the associations by comparing two regions with large differences in duration of time since the Khmer Rouge reign.

## Materials and Methods

A newly conducted structured interview survey performed in four regions in Cambodia (the provinces of Battambang and Banteay Meanchey in the north-west and Prey Veng and Svay Rieng in the south-east) collected information on trauma exposure, psychiatric symptoms, self-assessed health and socio-economic status. The south-east provinces were the first provinces in Cambodia to be liberated from Khmer Rouge control in 1979 by the invading Vietnamese military forces, while the north-west was under Khmer Rouge control until 1998. Although evidence shows a heightened incidence of exposure to trauma also under Vietnamese occupation (1978–90), this was much lower than during the Khmer Rouge era [5]. It was therefore assumed that exposure to trauma would be lower in the south-east than in the north-west, as well as the burden related to exposure considering the longer recovery period. The survey intentionally targeted rural districts where financial worries might be relatively more prevalent, in order to examine the contribution of past trauma on current economic status. Within these districts, communes, villages and respondents were randomly sampled [6]. The four provinces are in the middle range concerning household poverty, with around 30% of households below the poverty line in 2004 [13].

Individuals were eligible for inclusion if they were permanent residents of Cambodia aged 18–60, i.e. men and women of working age. Standardised structured interviews were conducted from November 28, 2011 to May 4, 2012 by eight trained psychiatric residents from the University of Health Sciences, Phnom Penh. Interviewers were gender-matched to the participants. Interviews were conducted in Khmer, with no one else in the household present except for the participant and lasted for 30–40 minutes. Interviewers read aloud the questions from the protocol. A supervisor in the field conducted quality control procedures, by monitoring members of the team and checking the completed interviews. The total sample consisted of 3200 persons; 1499 in the south-east region and 1701 in the north-west. The data collection (including consent procedure) and study was approved by the Swedish Regional Board of Ethics, Lund University, Sweden and by the Cambodian National Ethical Committee for Health Research, Phnom Penh. Before the start of the interview, the interviewers read the instructions (standardized letter) to the participants and the participants signed a separate consent form indicating that they had understood the purpose of the study, that participation was voluntary, and that their responses were anonymous. More detailed information on the data collection is available elsewhere [6].

### Exposure to trauma

Trauma exposure was assessed as two different types, i.e. conflict-related trauma and civilian trauma, in order to separate the effect of the atrocities committed mainly during the Khmer Rouge era from trauma occurring in family and work situations. As these two types of trauma differ, they may potentially have differing impact on economic status. Moreover, civilian trauma would be more relevant for those born after the Khmer Rouge era. Conflict-related trauma was assessed using the Harvard Trauma Scale [14], originally developed for evaluation of Cambodian survivors of mass violence. The focus of this scale is on trauma related to combat, war, and structural violence. A summary score on 28 trauma items was obtained for each individual to establish ever in life exposure. In addition to conflict-related trauma, civilian trauma was assessed as ever in life exposure to selected a priori chosen trauma items from the Composite International Diagnostic Interview (CIDI) [15]. These were adverse life events that were not directly related to mass violence during wartime or combat situations, e.g. life-threatening illness, exposure to natural disasters, childhood abuse etc. Similarly to conflict-related trauma, a summary score was obtained for affirmative response to 26 CIDI items. Events that had occurred during 1975–1979 were excluded from the civilian trauma score to reduce the possibility that civilian events might have been an effect of exposure to mass violence. Based on a median-split of the frequency distribution of the summary scores, exposure to conflict-related trauma was dichotomised as “high” (4 or more) vs. “low” (3 or less), and civilian trauma was dichotomised as “high” (2 or above) vs. “low” (0–1).

### Current health status

Current psychiatric status was assessed using the Cambodian version of the Hopkins Symptom Check List (HSCL) [16]. The 25 items are scored as to how much the person was bothered by that item during the past two weeks, i.e. 1 = not at all, 2 = a little, 3 = quite a bit, 4 = extremely. A total “psychiatric symptom score” was derived for each person, based on the total sum of the 25 items divided by the number of items answered. As suggested by Mollica et al. [16], the HSCL can also be used to assess “symptomatic depression” and “symptomatic anxiety”, as 10 of the 25 items represent anxiety and the remaining 15 items represent depression. A mean score >1.75 on the respective sub-scale items has been shown to indicate significant distress in Cambodia and Cambodian refugees [16] and would thus correspond to the presence of a symptomatic condition. These two measures, i.e. “symptomatic depression” and “symptomatic anxiety”, were combined into a single measure indicating “psychiatric co-morbidity”, thus representing the most severe level of symptomatic disability.

We also identified persons currently having an episode of “probable major depressive disorder” (PMDD), using an algorithm similar to that used by Bolton et al. [17]. Thus, a person was considered to have “probable major depressive disorder” if having HSCL symptoms on severity level 3 or 4 during the past 2 weeks, and having an indication of functional impairment based on the SF-36 subscale “limitations in usual role activities due to emotional health” [18], thereby meeting DSM-IV [19] criteria for major depressive disorder.

A brief screening scale for post-traumatic stress disorder (PTSD) was used to assess diagnostic status, consisting of 22 yes/no items from the CIDI diagnostic interview [15] corresponding to each of the DSM-IV [19] criteria for PTSD. Persons were categorised as “probable PTSD” (ever in life, currently during the past year, respectively) if affirmative responses indicated that they met the full DSM-IV criteria for an episode of at least one month’s duration during the specific time period.

In order to assess current stress due to daily life adversities, respondents were asked to rank on a scale of 1–4 (1 = never, 2 = occasionally, 3 = often, 4 = very often) how often they felt stressed in their daily lives by 12 items such as food scarcity, financial worry, and fear of landmine injury. For information on the development of the 12 item scale, see Cantor-Graae et al. [6]. A mean score was derived based on the summary scores divided by items answered. Persons were categorised as having “high” vs. “low” current stress, based on the median-split of the mean score frequency distribution.

The survey question on self-assessed health (“How would you describe your health?”) was derived from the SF-36 [18] with the standard five category outcomes. This measure was dichotomised as fair and poor vs. excellent, very good and good health.

### Economic status

Economic status was operationalised into three separate economic outcomes, two representing the household level (household debt and household wealth status) and one representing the individual level (economic inactivity). Presumably the individual effect is more direct, while the household effect might be more complex, due to the potential for compensation by other household members or, alternatively, the potential for spill-over effects.

Household debt was assessed as a yes/no answer to a question as to whether the household had any outstanding debts to other households or institutions. No information on the size of the possible debt was obtained. The measure was therefore used as a binary variable.

In correspondence with prior studies in low income countries, such as the Demographic and Health Survey (e.g. [20, 21]), a wealth index was also created as household (or personal) income is considered a poor measure of economic status in low income countries with large informal economies, subsidiary farming and home production. According to this strategy, a household’s relative wealth position in the sample can be considered an unobserved variable that is correlated with observable indicator variables. These indicator variables normally include assets, dwelling characteristics and land ownership [20]. Information was obtained on household assets during the interview, for the most part by direct observation. A factor analysis using principal factor procedure was conducted in order to reduce the number of variables (S1 Text) and to detect underlying relationships (the unobserved wealth status of the household). Following recommendations in Fabrigar et al [22], the number of retained factors is based on parallel analysis with a Scree-test as a sensitivity analysis. The oblique promax (3-power) rotation of factor loadings of the first factor was used to create the wealth variable as the correlation among factors exceeded the suggested threshold of 0.32 [22, 23]. This variable shows the households’ relative positions in the sample in terms of assets. The sample was then divided into quintiles (poorest, poor, middle, rich, and richest) [20] and into a binary variable indicating poverty status (see statistical analysis section).

Economic inactivity was based on the combined information derived from two questions in the survey; (1) self-reported main activity during the past 12 months where main activity refers to at least 6 months’ duration of the activity, and (2) self-reported employment status in the main occupation/economic activity. Based on these two questions, one measure was created where individuals were considered economically inactive if they reported mainly being unemployed, dependent or retired during the last year. All other subjects were included in the reference group, such as employed, students, and unpaid family workers, including farmers and home makers.

### Socio-demographic factors

Age was measured continuously while education was dichotomised into low vs. high education, where completion of middle school or higher was considered high education. This corresponds to more than six years of schooling. Marital status was dichotomised into married vs. unmarried where the former included co-habiting.

### Statistical analyses

Both household debt and economic inactivity were analysed using logistic regression. Also, the household wealth model was analysed using a logistic regression model instead of an ordered categorical model, as initial likelihood-ratio test of pairwise combination of the outcome categories [24] showed that the middle, rich and richest wealth quintiles were indistinguishable from each other based on the variables in the model. The wealth quintiles were therefore re-categorised into poor vs. non-poor in order to obtain more efficient estimates [24], which matches an often used cut-off point for relative poverty [20]. As the construction of the wealth index did not adjust for household size, this was controlled for in the wealth model using a household equivalence scale following Xu et al. [25].

All statistical analyses were conducted using STATA 13.1 [26] and the results are presented as odds ratios. The complex survey design was accounted for in all analyses using the “svy” prefix in STATA. Communes and villages were the primary and secondary sampling units, respectively, stratified by region (south-east and north-west). Weights were calculated as the inverse probability of being sampled. Variables were deemed significant at the 5% level although significance at 1 and 10% levels are also noted in the tables.

The three measures of economic status were used as separate dependent variables in the analyses. The independent variables of special interest were exposure to conflict and civilian trauma, respectively, and variables indicating poor mental health. Demographic and socio-demographic variables normally controlled for in this type of models were included in the models if available in the data. We endeavoured to have similar model specification for all models to facilitate comparison, although gender was not considered suitable for the models concerning household outcomes.

## Results

Descriptive statistics for the variables used in the statistical analyses are shown in Table 1. Persons in the south-east were somewhat older, better educated, and to a lesser extent living alone. Persons in the north-west showed poorer self-assessed health and higher prevalence of high civilian trauma exposure, PTSD, PMDD, current stress, and psychiatric co-morbidity, as well as higher psychiatric symptom scores. The two regions did not differ significantly in terms of economic outcomes.

**Table 1:** Descriptive statistics of the study sample.

	South-east (n = 1499)		North-west (n = 1701)		Total (n = 3200)	
*Continuous variables*						
	Mean	SD	Mean	SD	Mean	SD
Age	40.33	8.75	38.21	17.17	39.76	11.33***
Household size	2.37	0.41	2.50	0.74	2.40	0.52***
Psychiatric symptom score	1.22	0.22	1.40	0.55	1.27	0.32***
*Binary variables*						
	n	%	n	%	n	%
Women	755	50.27	855	48.22	1610	49.71
Low education	1018	68.20	1248	74.26	2266	69.85**
Single/unmarried	243	16.56	405	22.83	648	18.26***
Poor self-assessed health	835	55.00	1064	61.52	1899	56.77**
High conflict-related trauma	649	43.60	804	46.77	1453	44.46
High civilian trauma	527	32.22	884	51.54	1411	37.54***
Lifetime PTSD	17	0.93	123	6.94	140	2.56***
Current PTSD	11	0.66	40	2.29	51	1.10***
PMDD	27	1.43	74	4.30	98	2.21***
Psychiatric co-morbidity	55	3.46	176	9.66	231	5.15***
Current stress	451	27.99	1031	60.55	1482	36.82***
Household debt	799	54.21	1006	58.12	1805	55.27
Economically inactive	120	7.59	114	6.41	234	7.27
Household poverty status	637	45.59	659	38.93	1296	43.78

Design based Pearson chi2 tests were used except for continuous variables where Adjusted Wald tests were used.

Significance indicated at ** 5% and *** 1% level and indicates differences between the regions.

Tables 2 and 3 show the results concerning the relationship between trauma exposure and poor mental health and economic outcomes for the different models. Each separate economic outcome was analysed in relation to each separate trauma measure. For example, household debt was analysed separately for conflict-related trauma and civilian trauma. All models were adjusted for age, education, marital status and self-assessed health. The household poverty status regressions were also adjusted for household size, and the economic inactivity regressions were also adjusted for gender. The full results of Table 3 can be found as supporting information (S1–S3 Tables).

**Table 2:** Multivariate logistic regressions of different measures of trauma exposure in association with three economic outcomes, odds ratios and stratified by region.

	Household debt		Household poverty status		Economic inactivity	
	South-east	North-west	South-east	North-west	South-east	North-west
High conflict-related trauma	1.22	0.81	1.05	0.78	0.81	1.10
High civilian trauma	1.24	0.98	0.72**	0.96	0.99	0.84

Logistic regressions of each separate economic outcome are performed in relation to each separate trauma exposure measure, i.e. the models are not simultaneously adjusted for other measures of trauma exposure. All models are adjusted for age, education, marital status and self-assessed health. The household wealth regressions are also adjusted for household size while the economic inactivity regressions are also adjusted for gender.

Significance indicated at ** 5% level. SE = south-east region; NW = north-west region.

**Table 3:** Multivariate logistic regressions of different measures of trauma exposure/poor mental health in association with three economic outcomes, odds ratios.

	Household debt	Household poverty status	Economic inactivity
High conflict-related trauma	1.07	0.99	0.84
High civilian trauma	1.17	0.77**	0.89
Lifetime PTSD	1.19	1.25	1.43
Current PTSD	2.73*	3.21***	1.54
PMDD	2.26***	2.00**	4.38***
Psychiatric symptom score	2.85***	1.66***	2.62***
Psychiatric co-morbidity	2.45***	1.47**	2.68***
Current stress	1.49***	1.39**	1.50**

Logistic regressions of each separate economic outcome are performed in relation to each separate mental health measure, i.e. the models are not simultaneously adjusted for other measures of trauma exposure/poor mental health. All models are adjusted for age, education, marital status and self-assessed health. The household wealth regressions are also adjusted for household size while the economic inactivity regressions are also adjusted for gender.

Significance indicated at * 10%, ** 5%, and *** 1% level.

As shown in Table 2, high exposure to conflict-related trauma was not significantly associated with any economic outcome measure, either in the north-west or the south-east. That is, the difference in recovery period between the regions does not affect the results in the current sample. We therefore chose to pool the data from the two regions (Table 3). Civilian trauma however showed a negative association with poverty status in the pooled estimates. This effect is due to a strong negative effect in the south-east, while no association was found in the north-west. Of the two measures of PTSD, only current PTSD was associated with any economic outcome, and solely with poverty. PMDD was associated with more than twice the risk of household debt and poverty and more than four times the risk of being economically inactive. An increase in psychiatric symptom score as well as psychiatric co-morbidity was associated with increased risk of poorer economic outcomes in all models. Feeling stressed due to daily life adversities was also associated with increased risk of negative economic outcomes in all models although it showed the lowest effect size of all significant variables.

Poor self-assessed health was positively associated solely with household debt (S1–S3 Tables). Omitting self-assessed health status from the models had only very marginal effect on the estimates (results not shown). Low education, compared to higher education, was solely associated with an increased risk of poverty while being single was positively associated with risk of poverty and economic inactivity and negatively associated with household debt. Being a woman was strongly associated with a reduction in the risk of economic inactivity while age tended to show a U-shaped relation with economic inactivity only. Noteworthy is that self-assessed health and education were not associated with individual economic inactivity. The variable representing region was generally insignificant, as expected, although the results indicated that the risk of economic inactivity was lower in the north-west compared to the south-east (S3 Table).

There is a concern that the parallel analysis has led to overfactoring when constructing the wealth index. A sensitivity analysis was therefore run using a Scree-test as suggested by Fabrigar et al [22], which resulted in fewer factors retained. The results and interpretation were generally the same between analyses although the sensitivity analysis showed somewhat stronger associations, especially for low education. Civilian trauma, however, was no longer associated with poverty in the sensitivity analysis, and therefore should the negative association found in Table 3 be interpreted with caution.

## Discussion

This is the first study to investigate the association between high conflict-related and civilian trauma exposure and important economic outcome measures (household debt, poverty status, and economic inactivity) in a post-conflict, high-poverty setting. In addition, the study is based on the largest survey of trauma exposure and mental health thus far in Cambodia and purposely targeted two rural regions with different recovery time since the Khmer Rouge reign. We found no relationship between high conflict-related trauma exposure and any of the economic outcomes, whereas civilian-related trauma exposure was solely (negatively) associated with household poverty in the south-east of Cambodia. Current post-traumatic stress was related solely to poverty status while all other measures of mental health status were found to be strongly negatively associated with all measures of economic status.

The working hypothesis that trauma might affect the economic status of households and individuals beyond what might be mediated through poor mental health seems unlikely. High exposure to the traumatic events that, for example, occurred during the reign of the Khmer Rouge therefore did not seem to play an important role for current economic outcomes. The two regions studied have had different duration of exposure to conflict-related trauma and therefore different duration of recovery time since the Khmer Rouge regime (approximately 35 years in the south-east and 15 years in the north-west), although no difference in effect was shown. A mitigating effect over time of trauma impact has been shown in prior studies [14], which could serve as an explanation for the lack of association. However, the fact that exposure to civilian trauma, which is more recent, was also generally insignificant indicates that high lifetime exposure to trauma as such does not have an effect on economic status. The underlying reasons for the negative association found between civilian trauma exposure and household poverty status found in the south-east remain unknown. However, the impact of civilian trauma is likely to be modified by social structural and/or cultural factors, and regional differences in this regard may not have been captured in the current data. Also, our previous study did not find any negative impact of civilian trauma in the southeast on various aspects of mental health, in contrast to a significantly negative impact found in the northwest [6]. It remains to be explored to what extent factors promoting resilience to civilian trauma are particularly prominent in the southeast, and whether such factors could also be related to household wealth status. In addition, trauma exposure among Cambodian refugees in the USA was found to have a positive relationship with higher income, while the number of traumas was related to reduced income [10]. It is possible that a similar effect is evident here, although this cannot currently be studied.

Current measures of poor mental health were associated with negative economic outcomes whereas lifetime prevalence, i.e. ever in life PTSD, was insignificant. This indicates that the current mental health status is more relevant than general exposure to risk factors, suggesting that the focus of health policy in terms of poverty reduction should be on current mental health status rather than mental health history or exposure to trauma. It should be noted that current PTSD seemed to be the least important current measure of poor mental health in terms of economic outcomes. Very few respondents were categorised as having current PTSD, however, which potentially can explain the lack of significance. Current stress showed the smallest effect size of the significant variables. This measure is different from the others as it is not based on psychiatric symptoms. The lower effect size is, in this perspective, perhaps not surprising. It should also be noted that current stress and self-assessed health might be capturing some of the same “health” effect.

Self-assessed health was not associated with individual economic inactivity. Inactivity was measured over the last year and given the lack of social security systems in Cambodia, the individual presumably needs to continue to work also when faced with bad health. The fact that being a woman was strongly associated with reduced risk of economic inactivity is probably also an effect of the definition of economic inactivity, which excludes unpaid housework. Over 80% of the economically inactive individuals were men. Considering that domestic work in this context is mainly the responsibility of the women, it might be “easier” for women to remain active, given our definition, when faced with poor mental health. The economic inactivity measure therefore appears to capture the more severe cases, both in terms of self-assessed health and poor mental health. This was indeed expected, as we defined economic inactivity as primarily having to rely on others for support (due to minimal formal social security) [27], rather than being out of work but still in the labour force, i.e. a more formal measure of unemployment.

We explored the effect of trauma and poor mental health on economic status using three different outcomes, two on a household level and one on the individual level. Presumably the individual effect would be more direct, while the household effect might be more complex, due to the interplay between the individual and other household members. For example, a negative effect of trauma exposure on productivity could be compensated for by an increase in working hours by other family members. Alternatively, other family members may reduce work hours due to the need for home care of the trauma-exposed individual or even due to impaired health caused by spill-over effects [9]. It was therefore hypothesised that the individual and household level outcomes would differ, although the current study fails to find a clear distinction. It should be noted that the marital status variable also has the potential to capture household level effects (potential for compensation or spill-over). Being married was generally found to be protective with regard to poor economic outcome, except for household debt, potentially indicating a compensating effect.

The constructed wealth index functioned well in internal validation showing strong associations with ownership of various assets and household expenditures. However, the likelihood-ratio tests of whether pairs of the wealth categories can be combined indicated that several of the quintiles were indistinguishable from each other with respect to the variables included in the models. It was therefore considered appropriate to combine categories in order to increase the efficiency of the estimations, thus leading to a dichotomisation, i.e. “poor” (40%) vs. “rich” (60%). This is a higher poverty line compared to recent estimates of absolute poverty in Cambodia [13, 28] but it is in accordance with an often used cut-off point for relative poverty [20]. The dichotomisation was therefore reasonable from a statistical point of view and also seemed to capture the household poverty status level. In alignment with the current study’s results for economic inactivity, a prior study of Cambodian youths relocated to the USA found no effect of PTSD diagnosis on education and occupation [12]. Trauma has previously been found to be associated with employment status and earnings among Cambodian refugees in the USA [10], which is contrary to the current study. However, the definition of trauma differs, as Uba & Chung [10] focused on self-reported trauma that continued to affect the respondent. This difference in trauma definition and the fact that the study was performed within 10 years after the Khmer Rouge reign among Cambodians living in the USA can potentially explain the differences in results.

Both psychiatric symptom score and co-morbidity were independently related to economic status and were not a mediating effect of exposure to trauma. Although the current analytic strategy categorised persons in terms of the number of trauma experienced, individual vulnerability factors are strongly associated with the probability of developing mental health disorders following traumatic events (e.g. [29]). This is a probable reason why high trauma exposure as such does not have a negative role to play for economic outcomes in Cambodia, while current mental health status is so strongly implicated. That is, depending on personal vulnerability, not everyone who experienced trauma in the past is burdened by it today. Thus, the important aspect with regard to economic outcome seems to be the current mental health status per se [6].

It is important to note that the causality of the relationship cannot be established in the current study due to the cross-sectional design. Poor mental health could lead to poorer economic status just as well as financial difficulties and poverty could potentially be traumatic or cause traumatic events and poor mental health. For example, Rieder & Elbert [8] found an association between poverty and increased levels of family violence. This is especially a cause for concern, considering the positive relationship shown here between current stress and poor economic outcome. It is likely that the effect goes in both directions, i.e. that poor economic status also is stressful. This is further supported by the fact that financial worry was the most common daily stressor reported [6]. These results should therefore be interpreted with caution.

The number of individuals in the sample with poor mental health as defined by diagnostic criteria for various psychiatric disorders was relatively small, which may raise concerns regarding the robustness of the estimations. However, the use of more lenient definitions of psychiatric morbidity (total psychiatric symptom score, symptomatic anxiety and symptomatic depression) did not change the interpretation of the models. The relationship between poor mental health and poor economic outcomes found in the current study therefore appears valid.

It has been suggested that the more flexible nature of the job market in low income countries, compared to high income countries, including low skill jobs and less rigid work hours, could mitigate the negative effects of high trauma exposure and/or poor mental health [30]. This would imply that the negative consequences of high exposure to trauma and/or ill health have less effect on economic status in Cambodia, compared to high income countries, but also that differences in labour market even within a country could be expected to lead to differences in how mental health problems affects economic outcomes. It is common in the north-west to cross the border to work in Thailand. Having access to a greater number of job opportunities could thus allow the worst off individuals to remain economically active, while the same group in the south-east might suffer marginalisation, which potentially could explain the indication of a reduced risk of economic inactivity in the north-west.

Previous studies have shown that mental health disorders are linked to reduced productivity [27, 31]. Mental health interventions have also been shown to improve the individual’s economic outcomes in low- and middle-income countries [9]. However, there is no conclusive evidence of poverty alleviation having a positive effect on mental health, although the area is under-researched [9]. The positive association between poor mental health and poor economic outcomes, as found in the current study, suggests that effective interventions to improve mental health in Cambodia could potentially also improve the economic status of households and individuals, but greater efficacy is likely to be achieved by targeting current mental health status than previous trauma exposure.

The current study has some limitations. It is based on a cross-sectional data material, allowing no inferences to be drawn concerning causality, as discussed above. The duration of residence in the specific regions is unknown, and thus the effect of in- and out-migration cannot be determined. Chopra [27] suggests that workers in low income countries are likely to continue to work despite mental health problems due to absence of a welfare system. If this is true both for trauma exposure and poor mental health, it would indicate that only the severe cases are economically inactive, as discussed above. The measurements used for trauma exposure in the current study are based on a summation of the traumatic events and do not assess the severity (or frequency) of any given trauma item. Thus, the inability to distinguish between “severe” cases and “less severe” might not be optimal for capturing a negative effect of trauma on certain types of economic outcomes. A related issue is the risk of a survivor bias, i.e. that the worst off individuals, especially in terms of conflict-related trauma, have an increased mortality rate. The current sample would then represent less severely exposed persons, and thus potentially underestimate the association between trauma and economic outcomes. Further, our measure of trauma is divided into high versus low exposure to traumatic events. The underlying assumption is that the more traumatic events the individual is exposed to, the worse off the individual is. Although a dose-response effect has been shown in prior research [14], the validity of this assumption can be questioned. It is plausible that an individual exposed to one particularly horrifying traumatic event can be worse off, all else being “equal”, than an individual with several less severe traumatic events. Unfortunately, it is not possible to weight the traumatic events reported in this study based on severity in any objective manner. It should also be noted that the timing of the traumatic events within each measure is unknown. Finally, it is difficult to measure income and productivity in contexts such as rural Cambodia, and each measure is surrounded by uncertainty. We have approached this by using three different types of economic outcomes measured in three different ways. Since the results are similar among measures, we find that the separate results support the general conclusions regarding the association between, on the one hand, trauma and poor mental health and, on the other, economic status.

## Conclusion

This is the first study to use the most recent data from Cambodia to study the relationship between trauma/poor mental health and economic status. Based on the results, it appears that high exposure to trauma does not have a negative role to play for economic status in rural Cambodian households and individuals. Current mental health status, however, has a very strong association with economic status. Therefore, strategies that focus on current mental health status will probably have a higher chance of promoting economic status and reducing poverty in Cambodia than those focusing on previous exposure to trauma. It is likely that improvement in mental health is an important factor for poverty reduction as the negative labour market effects of mental health problems are largely avoidable.

## Supporting Information

S1 Table Multivariate logistic regressions on the association between high vs. low trauma exposure and mental health status and household debt, odds ratios. (DOCX)

S2 Table Multivariate logistic regressions on the association between high vs. low trauma exposure and mental health status and household poverty status, odds ratios. (DOCX)

S3 Table Multivariate logistic regressions on the association between high vs. low trauma exposure and mental health status and economic inactivity, odds ratios. (DOCX)

S1 Text Variables included in the factor analysis. (DOCX)
