# Supplementary material for: Trauma and Poor Mental Health in Relation to Economic Status: The Case of Cambodia 35 Years Later
Source: PLoS One. 2015 Aug 24;10(8):e0136410. doi: 10.1371/journal.pone.0136410 (PMC4547808; doi:10.1371/journal.pone.0136410)
Supplement: S1 Table — (DOCX) [file pone.0136410.s001.docx]

**Table S1. Multivariate logistic regressions on the association between high vs. low trauma exposure and mental health status and household debt, odds ratios.**

|  | I | II | II | IV | V | VI | VII | VIII |
| --- | --- | --- | --- | --- | --- | --- | --- | --- |
| North west region | 1.15 | 1.12 | 1.15 | 1.14 | 1.14 | 0.97 | 1.10 | 1.03 |
| Age | 1.05 | 1.05 | 1.06 | 1.06 | 1.05 | 1.04 | 1.05 | 1.05 |
| Age squared | 1.00* | 1.00* | 1.00* | 1.00* | 1.00* | 1.00 | 1.00* | 1.00 |
| Low education | 1.32* | 1.32* | 1.31* | 1.31* | 1.31* | 1.28* | 1.31* | 1.27 |
| Single/unmarried | 0.68* | 0.68* | 0.68** | 0.67** | 0.67** | 0.63** | 0.65** | 0.67** |
| Poor self-assessed health | 1.46*** | 1.46*** | 1.46*** | 1.48*** | 1.44*** | 1.30** | 1.43*** | 1.33** |
| High conflict-related trauma | 1.09 |  |  |  |  |  |  |  |
| High civilian trauma |  | 1.17 |  |  |  |  |  |  |
| Lifetime PTSD |  |  | 1.19 |  |  |  |  |  |
| Current PTSD |  |  |  | 2.73* |  |  |  |  |
| PMDD |  |  |  |  | 2.26*** |  |  |  |
| Psychiatric symptom score |  |  |  |  |  | 2.85*** |  |  |
| Psychiatric co-morbidity |  |  |  |  |  |  | 2.45*** |  |
| Current stress |  |  |  |  |  |  |  | 1.49*** |
| Constant | 0.41 | 0.40 | 0.40 | 0.40 | 0.42 | 0.17** | 0.45 | 0.42 |

Significance indicated at * 10%, ** 5%, and *** 1% level.
