# Supplementary material for: Trauma and Poor Mental Health in Relation to Economic Status: The Case of Cambodia 35 Years Later
Source: PLoS One. 2015 Aug 24;10(8):e0136410. doi: 10.1371/journal.pone.0136410 (PMC4547808; doi:10.1371/journal.pone.0136410)
Supplement: S2 Table — (DOCX) [file pone.0136410.s002.docx]

**Table S2. Multivariate logistic regressions on the association between high vs. low trauma exposure and mental health status and household poverty status, odds ratios**.

|  | I | II | III | IV | V | VI | VII | VIII |
| --- | --- | --- | --- | --- | --- | --- | --- | --- |
| North west region | 0.74 | 0.78 | 0.73 | 0.72 | 0.72 | 0.67* | 0.72 | 0.66* |
| Age | 1.04 | 1.05 | 1.04 | 1.04 | 1.04 | 1.04 | 1.04 | 1.04 |
| Age squared | 1.00 | 1.00 | 1.00 | 1.00 | 1.00 | 1.00 | 1.00 | 1.00 |
| Low education | 1.91*** | 1.89*** | 1.91*** | 1.91*** | 1.91*** | 1.89*** | 1.91*** | 1.86*** |
| Single/unmarried | 1.62*** | 1.61*** | 1.62*** | 1.61*** | 1.61*** | 1.56*** | 1.60*** | 1.62*** |
| Household size | 0.64*** | 0.64*** | 0.64*** | 0.63*** | 0.64*** | 0.63*** | 0.64*** | 0.63*** |
| Poor self-assessed health | 1.11 | 1.11 | 1.11 | 1.12 | 1.09 | 1.04 | 1.09 | 1.02 |
| High conflict-related trauma | 0.99 |  |  |  |  |  |  |  |
| High civilian trauma |  | 0.77** |  |  |  |  |  |  |
| Lifetime PTSD |  |  | 1.25 |  |  |  |  |  |
| Current PTSD |  |  |  | 3.21*** |  |  |  |  |
| PMDD |  |  |  |  | 2.00** |  |  |  |
| Psychiatric symptom score |  |  |  |  |  | 1.66*** |  |  |
| Psychiatric co-morbidity |  |  |  |  |  |  | 1.47** |  |
| Current stress |  |  |  |  |  |  |  | 1.39** |
| Constant | 0.79 | 0.77 | 0.81 | 0.83 | 0.86 | 0.58 | 0.87 | 0.86 |

Significance indicated at * 10%, ** 5%, and *** 1% level.
