# Supplementary material for: Trauma and Poor Mental Health in Relation to Economic Status: The Case of Cambodia 35 Years Later
Source: PLoS One. 2015 Aug 24;10(8):e0136410. doi: 10.1371/journal.pone.0136410 (PMC4547808; doi:10.1371/journal.pone.0136410)
Supplement: S3 Table — (DOCX) [file pone.0136410.s003.docx]

**Table S3. Multivariate logistic regressions on the association between high vs. low trauma exposure and mental health status and economic inactivity, odds ratios.**

|  | I | II | III | IV | V | VI | VII | VIII |
| --- | --- | --- | --- | --- | --- | --- | --- | --- |
| North west region | 0.71 | 0.73 | 0.68* | 0.70 | 0.64* | 0.55** | 0.64* | 0.60** |
| Women | 0.13*** | 0.13*** | 0.14*** | 0.14*** | 0.13*** | 0.12*** | 0.13*** | 0.13*** |
| Age | 0.84*** | 0.84*** | 0.84*** | 0.84*** | 0.83*** | 0.83*** | 0.83*** | 0.83*** |
| Age squared | 1.00*** | 1.00*** | 1.00*** | 1.00*** | 1.00*** | 1.00*** | 1.00*** | 1.00*** |
| Low education | 1.07 | 1.07 | 1.07 | 1.07 | 1.08 | 1.08 | 1.09 | 1.06 |
| Single/unmarried | 2.29** | 2.25** | 2.22** | 2.24** | 2.23** | 2.11** | 2.12** | 2.24** |
| Poor self-assessed health | 1.57 | 1.57 | 1.56 | 1.57 | 1.55 | 1.48 | 1.57 | 1.38 |
| High conflict-related trauma | 0.84 |  |  |  |  |  |  |  |
| High civilian trauma |  | 0.89 |  |  |  |  |  |  |
| Lifetime PTSD |  |  | 1.43 |  |  |  |  |  |
| Current PTSD |  |  |  | 1.54 |  |  |  |  |
| PMDD |  |  |  |  | 4.38*** |  |  |  |
| Psychiatric symptom score |  |  |  |  |  | 2.62*** |  |  |
| Psychiatric co-morbidity |  |  |  |  |  |  | 2.68*** |  |
| Current stress |  |  |  |  |  |  |  | 1.50** |
| Constant | 0.66 | 0.76 | 0.81 | 0.78 | 0.92 | 0.37 | 1.00 | 0.81 |

Significance indicated at * 10%, ** 5%, and *** 1% level.
