# Supplementary material for: Trauma and Poor Mental Health in Relation to Economic Status: The Case of Cambodia 35 Years Later
Source: PLoS One. 2015 Aug 24;10(8):e0136410. doi: 10.1371/journal.pone.0136410 (PMC4547808; doi:10.1371/journal.pone.0136410)
Supplement: S1 Text — (DOCX) [file pone.0136410.s004.docx]

**Text S1. Variables included in the factor analysis.**

The following binary variables were included in the factor analysis: radio, TV, phone, cell phone, video/DVD, stereo, camera, satellite dish, animal for transportation, motorcycle, car, household has transport, sewing machine, stove, electric iron, electric fan, suitcases, generator, batteries, uncommon household equipment, canvas mat, sofa set, dining set, bed set, wardrobe, computer, animal cart for production, tractor, plough, thresher, hand tools, water pump, uncommon water and agriculture production means, and large dwelling size. Assets with few positive observations or very low variance were not included. Likewise, education and occupation were not included in order to create a wealth index that represents the economic status of the household, and not the socioeconomic status [[20](#_ENREF_20)].
